# Remote acoustic sensing as a safety mechanism during exposure of metal implants to alternating magnetic fields

**DOI:** 10.1371/journal.pone.0197380

**Published:** 2018-05-10

**Authors:** Bingbing Cheng, Yonatan Chatzinoff, Debby Szczepanski, Chenchen Bing, Sumbul Shaikh, Omar Wyman, Cameron E. Perry, James A. Richardson, Dennis K. Burns, Bret M. Evers, David E. Greenberg, Rajiv Chopra

**Affiliations:** 1 Department of Radiology, UT Southwestern Medical Center, Dallas TX, United States of America; 2 Department of Internal Medicine, UT Southwestern Medical Center, Dallas TX, United States of America; 3 Department of Pathology, UT Southwestern Medical Center, Dallas TX, United States of America; 4 Department of Microbiology, UT Southwestern Medical Center, Dallas TX, United States of America; 5 Advanced Imaging Research Center, UT Southwestern Medical Center, Dallas, TX, United States of America; University of Illinois at Urbana-Champaign, UNITED STATES

## Abstract

Treatment of prosthetic joint infections often involves multiple surgeries and prolonged antibiotic administration, resulting in a significant burden to patients and the healthcare system. We are exploring a non-invasive method to eradicate biofilm on metal implants utilizing high-frequency alternating magnetic fields (AMF) which can achieve surface induction heating. Although proof-of-concept studies demonstrate the ability of AMF to eradicate biofilm in vitro, there is a legitimate safety concern related to the potential for thermal damage to surrounding tissues when considering heating implanted metal objects. The goal of this study was to explore the feasibility of detecting acoustic emissions associated with boiling at the interface between a metal implant and surrounding soft tissue as a wireless safety sensing mechanism. Acoustic emissions generated during in vitro and in vivo AMF exposures were captured with a hydrophone, and the relationship with surface temperature analyzed. The effect of AMF exposure power, surrounding media composition, implant location within the AMF transmitter, and implant geometry on acoustic detection during AMF therapy was also evaluated. Acoustic emissions were reliably identified in both tissue-mimicking phantom and mouse studies, and their onset coincided with the implant temperature reaching the boiling threshold. The viscosity of the surrounding medium did not impact the production of acoustic emissions; however, emissions were not present when the medium was oil due to the higher boiling point. Results of simulations and in vivo studies suggest that short-duration, high-power AMF exposures combined with acoustic sensing can be used to minimize the amount of thermal damage in surrounding tissues. These studies support the hypothesis that detection of boiling associated acoustic emissions at a metal/tissue interface could serve as a real-time, wireless safety indicator during AMF treatment of biofilm on metallic implants.

## 1. Introduction

In the United States, over one million total knee and hip replacement procedures are currently performed annually [1]. These numbers are expected to increase by up to 600% in the next decade due to current population and health trends [2–4]. Although the risk of developing a post-surgical prosthetic joint infection (PJI) is low (approximately 1–2% [5]), the treatment of these infections is prolonged, painful, and expensive. Currently the gold standard for treating PJI in the US is a two-stage revision arthroplasty [6]. The infected joint is removed and replaced with a spacer in a first surgery, followed by weeks of antibiotic administration to completely eradicate the infection. Then a new prosthesis is implanted in a second surgery [6]. The projected economic burden on the US healthcare system from PJI by 2020 is $1.6 billion [7].

A major factor preventing resolution of PJI with traditional antibiotic therapy is the formation of biofilm on the prosthetic surface. Biofilm is a thin (less than 1 mm) film of extracellular polymeric substances (EPS) produced by a microorganism allowing aggregation and adhesion onto the implant surface [8–10]. Biofilm shields bacteria and persister cells from their environment [11], compromising the effectiveness of both antibiotics and the immune response [12,13].

Our group and others have recently proposed a non-invasive method to eradicate biofilm on infected metal prostheses utilizing alternating magnetic fields (AMFs) [14–16]. When metallic prostheses are exposed to a high-frequency AMF, electrical currents referred to as eddy currents are induced on the outer surface. Rapid heating induced by these currents can be achieved on the metal surface within a thin layer (micrometers to millimeters), referred to as the skin depth [17]. The skin depth depends on the AMF frequency, and is on the order of 1 mm in stainless steel for a 100 kHz AMF [17]. As a result, rapid superficial heating where the biofilm resides is achievable and can be used to selectively destroy the target pathogen.

Although proof-of-concept studies demonstrate the ability of AMF to eradicate biofilm in vitro [14,15], there is a legitimate safety concern of potential thermal damage to surrounding tissues when considering heating implanted metal objects. This risk of thermal damage does not arise from direct AMF heating of tissues when the applied AMF has a relatively low frequency (hundreds of kHz), but rather heat conduction into tissue from the heated implant. However, direct tissue heating from AMF becomes more significant at higher frequencies (above 1 MHz). Compared to metal, AMF has little to no interaction with soft tissues. However, if the metal implant is overheated, there might be some tissue damage generated around the implant due to thermal conduction. Furthermore, since AMF exposures are intended to be delivered non-invasively, it is desirable to monitor the temperature of the implant non-invasively as well. MR thermometry is the only clinical non-invasive method capable of monitoring tissue temperatures, however, it only measures the temperature of soft-tissue, and does not work well in the presence of magnetic susceptibility variations as would be encountered around a metal implant.

The objective of this study was to investigate a method for remote safety monitoring during AMF therapy. The mechanism explored was the detection of acoustic emissions associated with boiling at the interface between a metal implant and surrounding soft tissue as a wireless safety sensing mechanism. To prove the feasibility of this technique, a proof-of-concept AMF system was built with the ability to simultaneously deliver AMF to metal implants and to detect sound signals emitted from the surface. Acoustic emissions generated during in vitro and in vivo AMF exposures were recorded with a hydrophone and analyzed, and the relationship with surface temperature was evaluated. The effects of AMF power, surrounding media, implant position, and implant geometry were also investigated. To evaluate safety, the extent of thermal damage to tissues surrounding a metal implant was assessed in vivo and compared with mathematical simulations. Finally, a human-scale AMF system with this safety sensing mechanism was constructed, and the feasibility of heating a human knee implant was evaluated.

## 2. Results

### 2.1 System and data analysis

**Fig 1:**
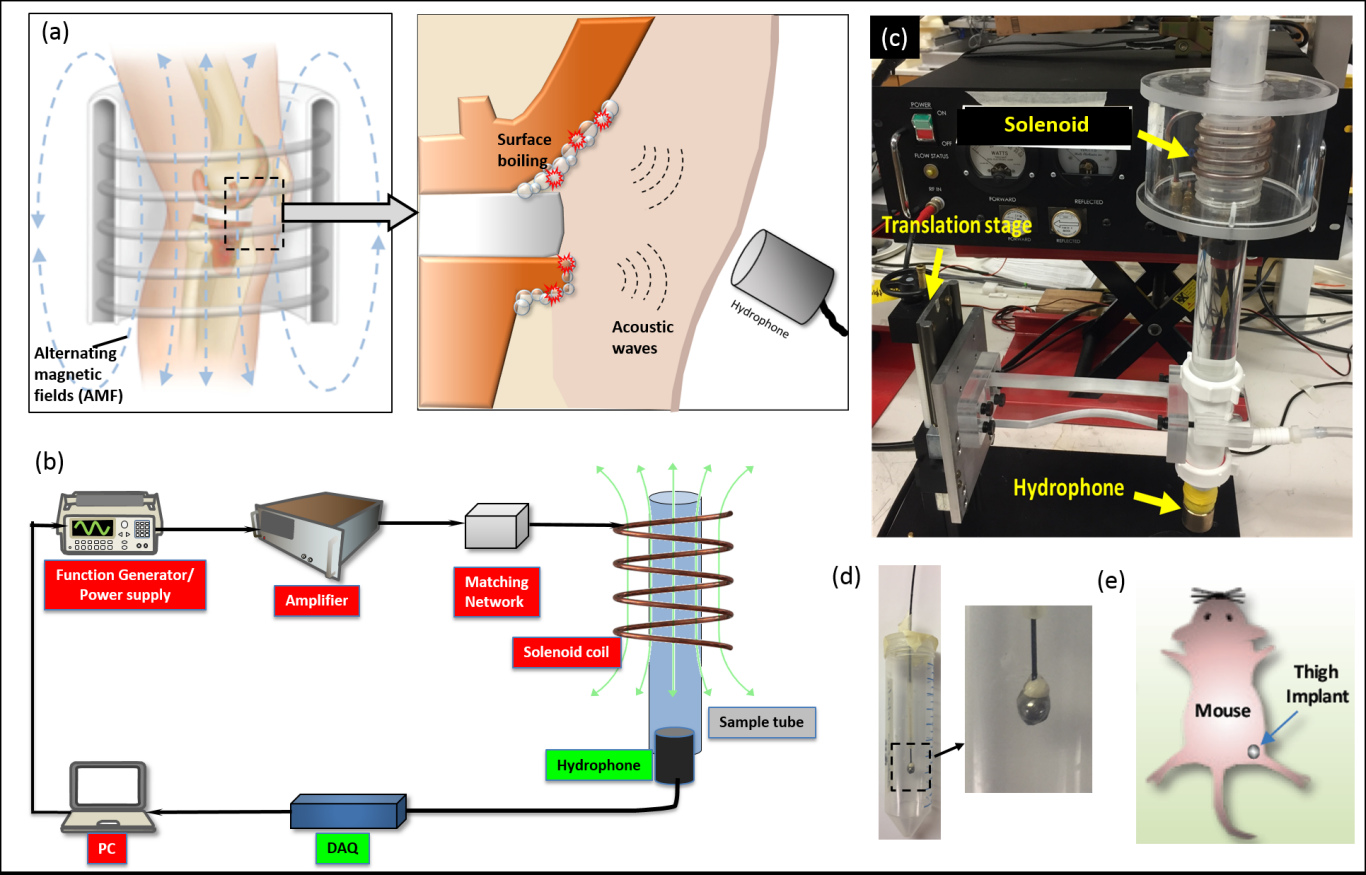
AMF safety concept and the experimental systems used to evaluate it. (a) A conceptual diagram depicts the AMF application and the mechanism of acoustic detection. Localized heating that exceeds the threshold for boiling will generate acoustic energy that can be detected with a receiver from outside the body. (b) A schematic diagram of the system, which consists of two parts: 1) AMF subsystem for delivery of AMF to objects in the coil, including a function generator/power supply, a radio-frequency amplifier, an impedance matching network and a custom-built solenoid coil; and 2) Acoustic subsystem to detect boiling-associated acoustic signals at the implant-tissue interface, including a hydrophone, and a data acquisition card. The metal sample was placed in the sample tube surrounded by water; (c) A photograph of the AMF system; (d) The metal ball bearing phantom model. The conical tube can be filled with tissue-mimicking phantom, hydrogel, oil or other materials. (e) The metal ball bearing mouse model. A 4.8 mm metal ball was embedded in the thigh muscle.

Fig 1A shows the concept of utilizing remote acoustic detection during AMF treatment. When a metal implant is exposed to high-frequency alternating magnetic fields, its surface will be rapidly heated from induced eddy currents. When any point on the surface reaches approximate 100°C, localized boiling of the implant-encapsulating liquid medium will occur, causing the formation and collapse of bubbles at the interface between the metal implant and surrounding environment. This activity will immediately generate acoustic waves propagating outward from the location of boiling, which can be detected by a remote acoustic sensor, such as a hydrophone. These acoustic emissions can be used as an indicator to identify that a location on the implant has been heated to approximate 100°C. The indicator can be used to shut off the AMF system to avoid heating beyond this temperature.

A system was developed for in vitro and animal studies to evaluate the feasibility of this concept (Fig 1B and 1C). The system comprises two components: 1) an AMF subsystem to heat metal implants and 2) an acoustic subsystem to detect sound emissions. The AMF subsystem included a function generator, radiofrequency amplifier, impedance matching network, and solenoid coil. The system was operated at 500 kHz to achieve a skin depth of approximately 600 μm. The acoustic subsystem included a hydrophone and a data acquisition card (DAQ). Both subsystems were controlled by a lab computer using custom-written software in Labview. Fig 1C is a photo of the major components of the AMF system. A hydrophone was attached to a sample tube placed inside the solenoid via a 50 cm long cylinder of fluid. Metal implants either embedded in phantoms (Fig 1D) or mice (Fig 1E) were placed inside the center of the solenoid for AMF exposures. Fig 1D and 1E show the phantom and animal models which were utilized in this study. In the phantom model, a 4.8 mm diameter stainless steel ball was embedded in the center of a 50 ml conical tube. The tube was filled with different media, including tissue-mimicking phantoms, hydrogels, or motor oil. A non-magnetic fiber-optic temperature sensor was attached to the ball to monitor its temperature change during AMF exposures. For the animal model, the same size stainless steel ball was surgically implanted into the thigh muscle of the mice.

**Fig 2:**
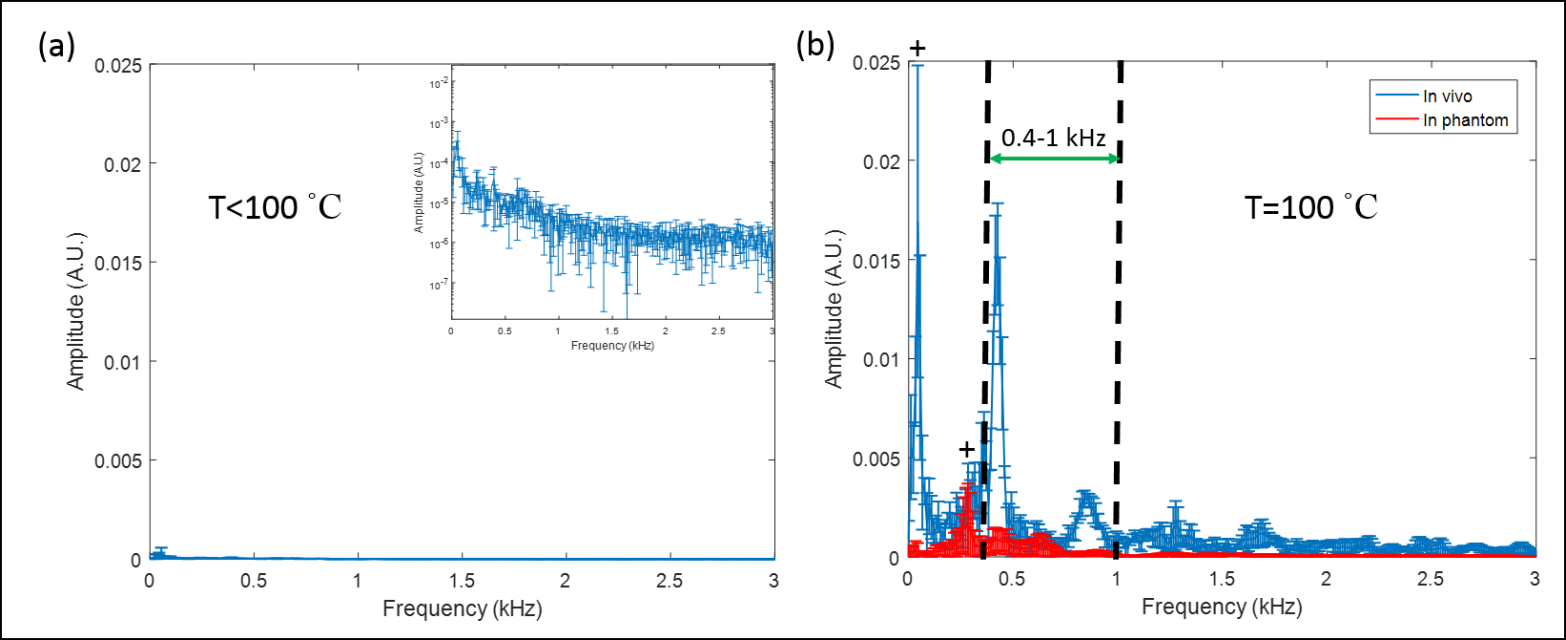
Frequency spectrum of the sound signals acquired from the hydrophone. (a) Frequency spectrum with no boiling occurrence in vivo (T<100°C, n = 3). The inset is the same data shown in the log scale. (b) Frequency spectrum at the onset of boiling (T = 100°C) both *in vivo* (n = 4) and in tissue-mimicking phantoms (n = 4); +: environmental noise. The figure shows that there are several consistent peaks between 0.4–1 kHz which are associated with boiling. These peaks were selected as boiling signatures and the area under curve (AUC) within 0.4–1 kHz was calculated to represent the sound signal strength. Statistics: mean ± std.

Fig 2 shows the frequency spectrum of the signal recorded by the hydrophone prior to and at the onset of boiling. In the absence of boiling (Fig 2A) the frequency spectrum shows little signal across the entire frequency range of 0–3 kHz, only several small peaks appeared in the low frequencies (< 400 Hz) which were considered as the environmental noise. Once boiling occurs, significant energy appears across the entire frequency spectrum with a concentration of energy at specific frequencies. The strength of the measured signal was consistently stronger in vivo compared to the tissue-mimicking phantom. In both cases the measured frequency spectrum was very repeatable across multiple phantoms and animals, shown by the error bars (standard deviation) of the frequency spectrums. Those peaks labeled with plus (+) signs arose from a combination of the boiling signal and environmental noise, such as noise from the fan (4 of them were built in the AMF system for electronic components cooling), water chiller, etc. Due to the presence of several major peaks between 400–1000 Hz, this frequency band was selected since it captured a significant portion of the energy while avoiding environmental noise. The area under the curve (AUC) of the frequency spectrum within this characteristic band was calculated continuously during AMF exposures to represent the sound signal strength. Once the AUC value exceeded a pre-determined threshold (i.e., 10 times the baseline AUC), it was assumed that boiling was present at the implant surface. The control software had the capability to turn off the AMF exposure once the boiling AUC threshold was detected.

### 2.2 Relationship between metal implant surface temperature and acoustic emissions

**Fig 3:**
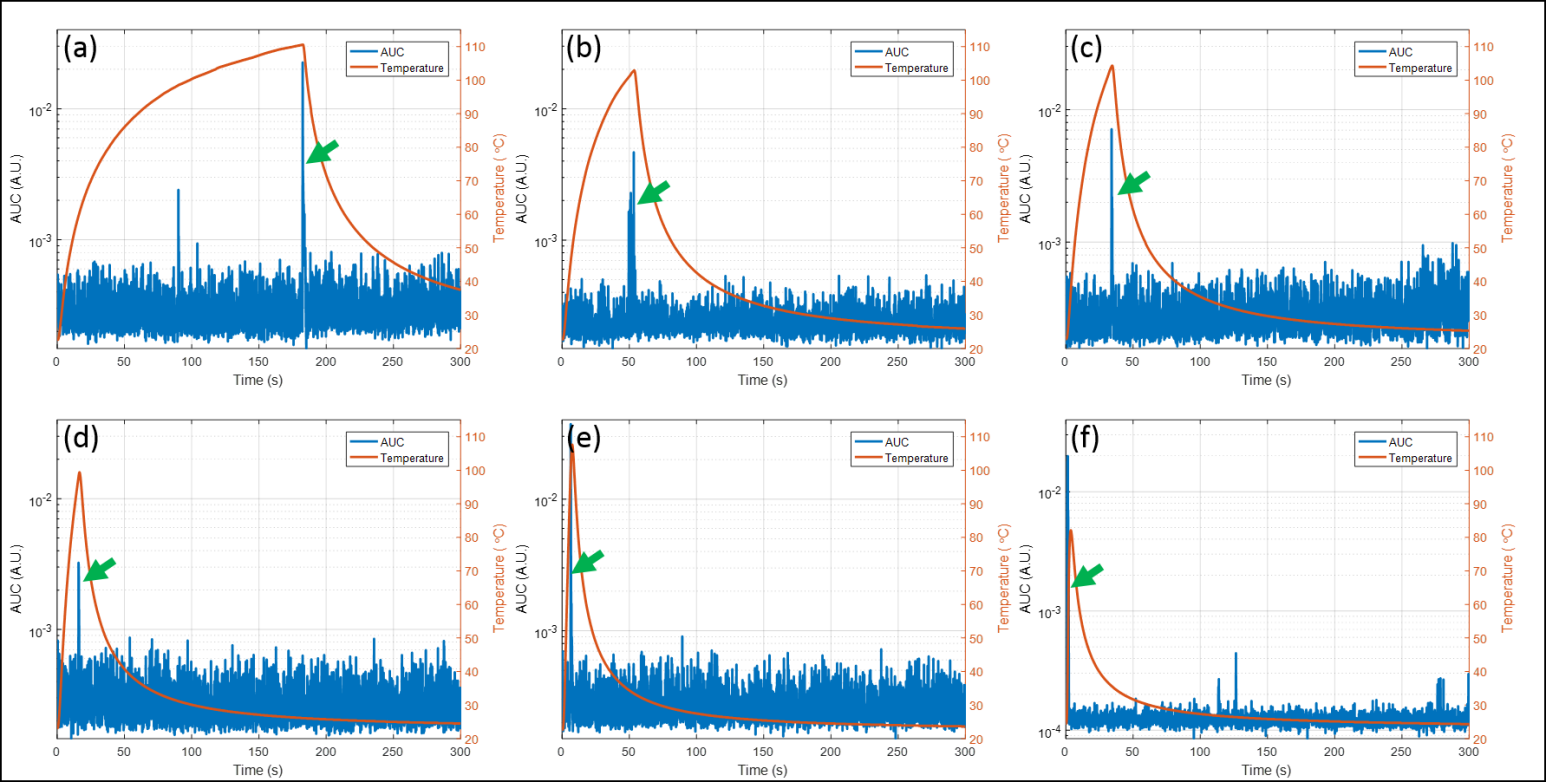
Relationship between temperature and AUC during AMF exposures of a ball bearing embedded in a tissue-mimicking phantom. The left y axis and blue curve represent the AUC of the acoustic signal and the right y axis and orange curve show the temperature of the ball. The green arrow indicates when a boiling signal was detected. The powers corresponding to (a)-(f) are 200, 250, 300, 400, 800, and 4300 W, respectively. The temperature was always approximately 100°C when boiling was detected. Furthermore, the time required to reach boiling significantly decreased with increasing power.

The relationship between the measured sound signal and metal surface temperature is shown in Fig 3. In this experiment, a 4.8 mm diameter stainless-steel ball embedded in a tissue-mimicking phantom was exposed to AMF at 6 different powers. The ball temperatures and acoustic emissions were measured simultaneously throughout the exposure. Fig 3A–3F show the results for 200, 250, 300, 400, 800, and 4300 W, respectively. The blue curves represent the AUC of the sound signal over time, and the orange curves represent the temperature of the metal ball during the AMF exposures. The green arrows indicate a sudden increase in AUC that occurred once the ball was heated to approximately 100°C. The average temperature when this increase in emissions was detected was 102.7 ± 4.2°C, confirming this was associated with the presence of boiling at the metal/phantom interface. These acoustic emissions were at least 10 times stronger than the baseline AUC. The signal to noise ratio (SNR) for the in vivo studies was even larger because the acoustic emissions when boiling was generated were stronger due to the possible higher water content surrounding the implant while the baseline AUC remained at the same level (Fig 2). The lower temperature at boiling shown in Fig 3F was likely due to the fact that heating occurred rapidly at 4300 W, thereby exceeding the response time of the fiber-optic sensors. Another aspect shown in Fig 3 is the ability to terminate heating based on acoustic emissions. System power was shut off when the AUC was greater than 0.003, and the temperature did not continue beyond the boiling point. This power shut-off ability existed for all tested conditions, even in the case of rapid heating at 4300 W.

### 2.3 Effect of media on boiling detection

**Fig 4:**
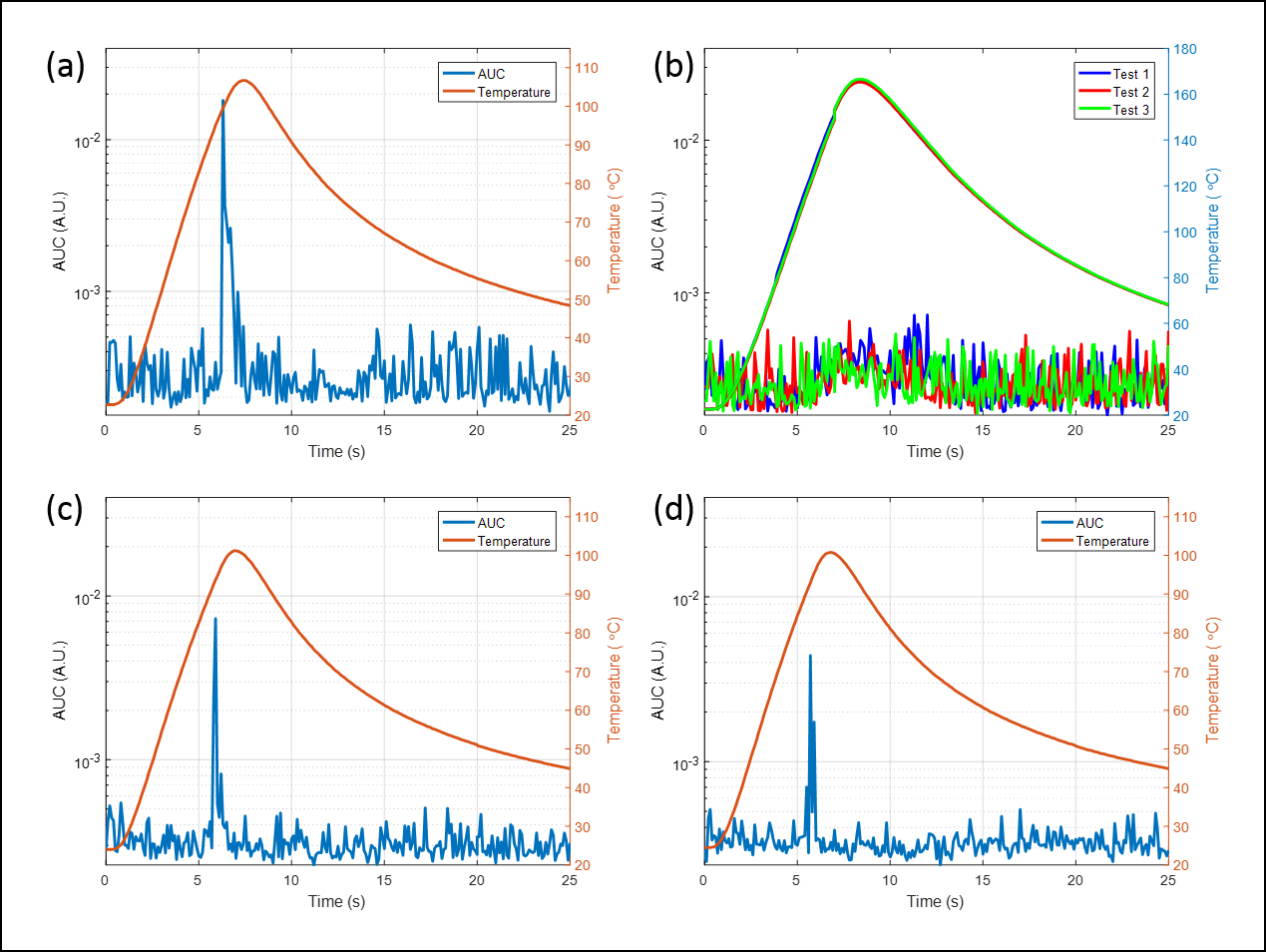
Boiling detection and temperature characterization of ball bearing in different media. (a) tissue-mimicking phantom; (b) motor oil; hydrogel with viscosities of (c) 1 mm^2^/s and (d) 528 mm^2^/s. Similar boiling signal and temperature curve were observed in (a), (c) and (d). However, since the boiling point of oil is much higher than 100°C, boiling was not detected even when temperature of the ball bearing reached 160°C.

Fig 4 shows the temperature profiles and acoustic emissions during 800 W AMF exposures of a metal ball embedded in different media including: a) a tissue-mimicking phantom, b) motor oil, and c-d) hydrogels of different viscosity. The viscosity of hydrogel in Fig 4C and 4D were 1 mm^2^/s and 528 mm^2^/s, respectively. The similar temperature profiles and acoustic emissions measured in Fig 4A, 4C and 4D suggest that AMF induction heating and boiling detection was insensitive to the composition of the surrounding water-contained media. Note that there is a temperature rise after the boiling was detected. This is because of the thermal diffusion from the boiling location to the sensor location since the sensor was not at the same place where boiling happened. However, the results of three independent tests in motor oil indicate that although the temperature profile of the metal ball was similar to the other media, no significant acoustic emissions were detected. This can be explained by the fact that the boiling point of oil is much higher than 100°C, thus the ball did not reach a temperature sufficient to generate acoustic emissions in this medium. Eventually boiling should occur in oil once the temperature reached the boiling point, however, heating was turned off manually at 160°C in order to protect the fiber-optic temperature sensors.

### 2.4 Effect of position on heating efficiency and boiling detection

**Fig 5:**
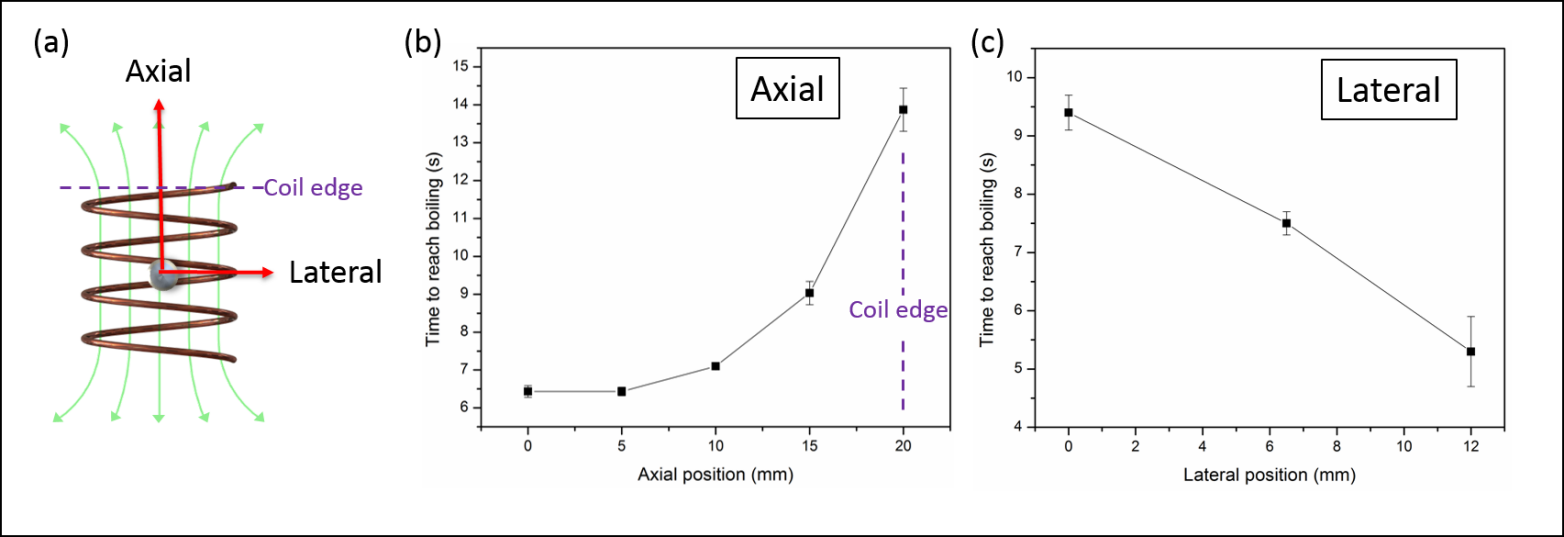
The influence of implant positioning within the coil on heating efficiency and time to boiling. (a) A diagram showing how a metal ball was moved in the axial and lateral direction from center towards the edge of the coil; (b) The relationship between the time to boiling and axial position; (c) The relationship between the time to boiling and lateral position. The experiments were conducted in low-viscosity hydrogel. Statistics: mean ± std (n = 3).

Fig 5A shows a schematic diagram of the experiment setup. A 4.8 mm stainless steel ball embedded in a hydrogel was placed in the center of the coil. The ball was moved in the axial and lateral directions and AMF exposures were performed at each location. Fig 5B shows the time required to reach boiling at different axial locations. The time to boiling increased exponentially (from 6.4 ± 0.2s to 13.9 ± 0.6s) as the ball was displaced from the coil center, due to a reduction in the heating efficiency. Fig 5C shows that when the ball was closer to the edge of the coil in the lateral position, the time to boiling became shorter, indicating a higher heating efficiency near the edge of the coil.

### 2.5 Relationship between time to boiling and AMF power in vitro and in vivo

**Fig 6:**
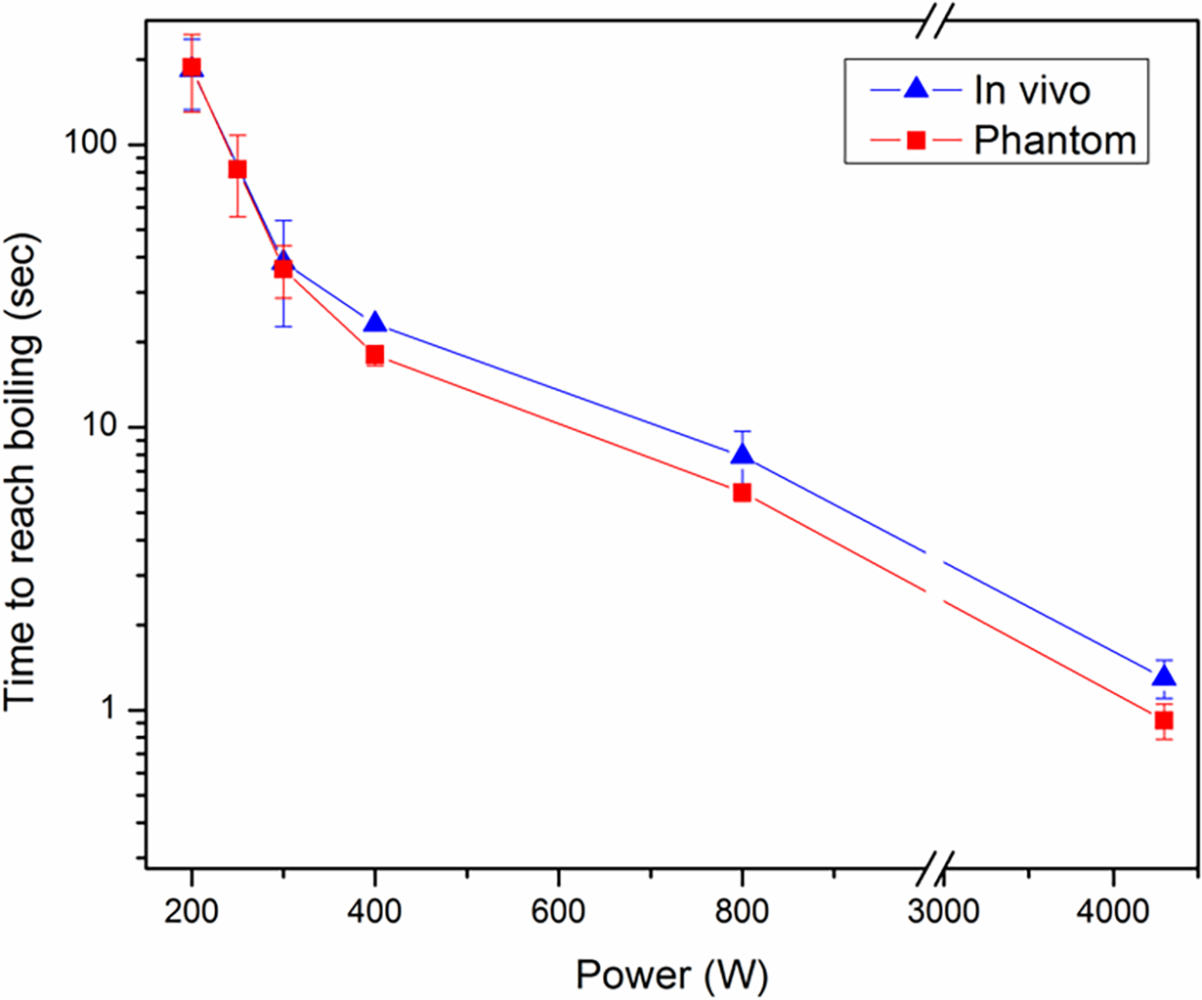
Time to boiling decreases with increasing AMF power in both tissue-mimicking phantom and in vivo. The red curve (squares) depicts the result for a ball embedded in a tissue-mimicking phantom, while the blue curve (triangles) depicts the result for the same ball embedded in the thigh muscle of mice. The relationship between time to boiling and power was similar for both experimental groups. Statistics: mean ± std (n = 4).

Fig 6 shows the time required to reach boiling for the ball implant as a function of AMF power both in tissue-mimicking phantoms and in the thigh muscle of mice. Both in vitro and in vivo results show that less time was required to heat the metal surface to 100 ˚C to reach boiling with higher power. For instance, the in vivo time to boiling was 184.7 ± 51.5s under AMF exposures of 200 W. However, the time was significantly reduced to 1.3 ± 0.2s when the power increased to 4300 W. This trend can be identified in both experimental groups. Furthermore, the time to boiling was slightly longer in vivo, which is expected due to the presence of tissue perfusion (which takes some portions of heat away during the exposures).

### 2.6 Effect of implant geometry on boiling detection

**Fig 7:**
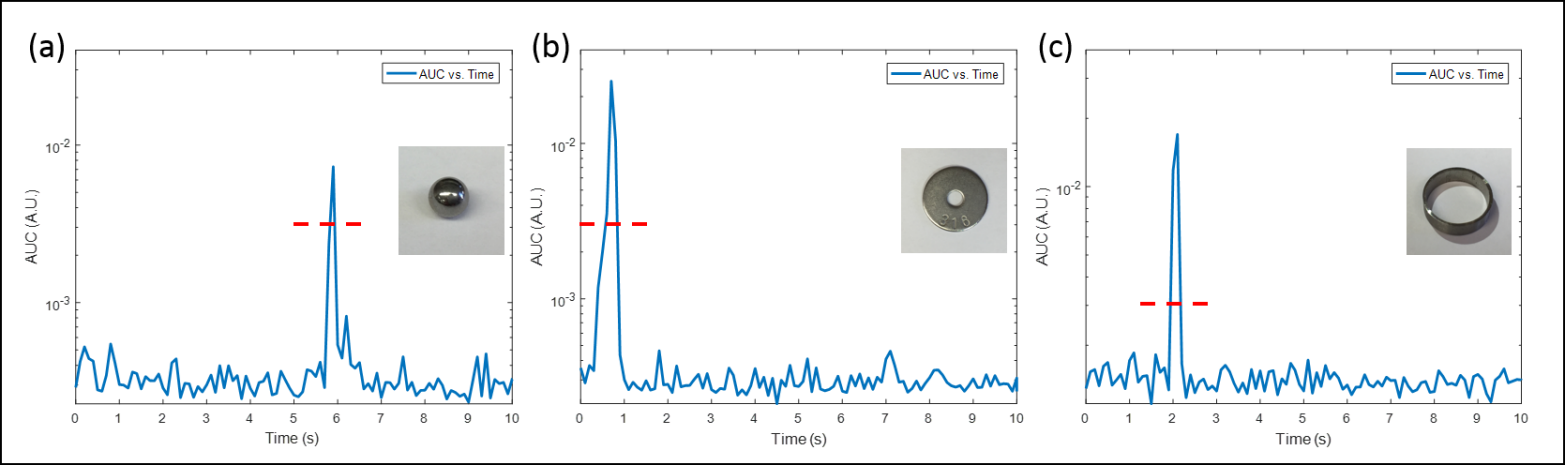
Boiling detection for implants with different geometry. (a) Ball; (b) Washer; and (c) Ring. The red dashed lines indicate the boiling signal detection threshold (0.003). Power utilized in the experiments: (a) 800, (b) 650, and (c) 650 W. The figure shows that boiling was detected for all implants irrespective of their geometry. The time to reach boiling was different however, due to the different heating efficiencies of each implant.

Fig 7 shows the acoustic emissions and feasibility of detecting boiling from three kinds of metal implants with different geometries: a ball bearing, washer, and ring. All of them were made of stainless steel. All the implants were embedded in a hydrogel with viscosity similar to water. Surface boiling was successfully detected for all of the three geometries, as shown in Fig 7. The red dashed line indicates the boiling AUC threshold. The time to achieve boiling of the ball bearing was 5.9 s for an 800 W AMF exposure. For the washer and ring, the time to boiling was 0.6 and 2 s respectively, for a 650 W AMF exposure. The differences in the power and time required to reach boiling are related to the geometry and the different current densities induced on the metal objects. The same input signal was fed to the amplifier, but because the different geometries had a different coupling with the solenoid and tank circuit, a different net output power was delivered to the implant in each case.

### 2.7 In vivo safety evaluation

**Fig 8:**
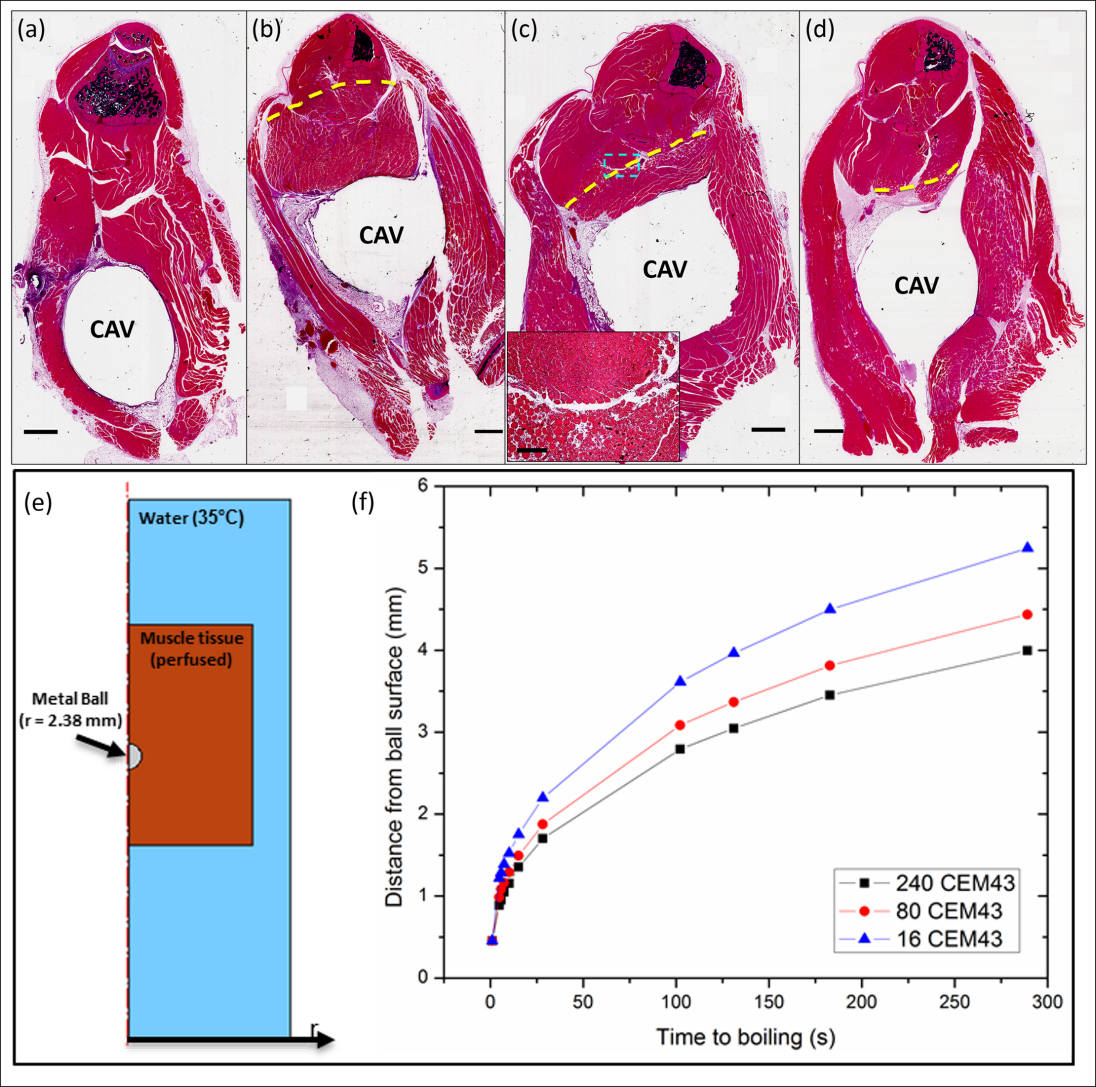
AMF exposures produce highly localized tissue damage in vivo. H&E-stained sections through the thighs of mice receiving AMF exposures. The cavity (CAV) represents the location of a surgically implanted 4.8 mm stainless steel ball. A sham-treated mouse (a) 7 days after surgical implantation shows a residual inflammatory response at the edge of the cavity. A mouse receiving a 190 W AMF exposure for 220 s (b) exhibits a rim of thermal damage surrounding the implant and extending approximately 3 mm from the cavity. The boundary between the damaged area and normal tissue is marked by a yellow-dashed line. A similar pattern of damage is seen for a mouse exposed to 800 W for 15 seconds (c), however the radial extent is only approximately 1.3 mm. A mouse after a 4300 W AMF exposure for 1.5 s (d) exhibits a circumferential pattern of damage extending to approximately 0.6 mm from the cavity rim. In all cases, the transition from damaged to normal muscle (dashed line in top panels) is abrupt. Scale bars represent 1 mm. To representatively show the details in the transition zone, a high-magnification image of the area indicated by a blue-dashed box in (c) was obtained and showed as the inset with the scale bar of 200 μm. (e-f) Numerical simulations of the mouse AMF exposures shows the relationship between AMF power, duration and thermal damage. The distance to the 240 CEM43 boundary (extent of irreversible thermal damage), the 80 CEM43 boundary (extent of reversible damage), and 16 CEM43 boundary (extent of initial bone damage) from the surface of the implanted metal ball are shown as a function of the time required to reach a surface temperature of 100°C on the ball. The construct of the 2D axisymmetric model is shown in e), while the trend for the damage contours is shown in f). The graph shows the rapidly reduced thermal damage distance as the time to boiling is decreased due to the increasing AMF power, indicating the enhanced safety of high power short AMF exposures. In addition, the distance between the black and red curves decreases as the time to boiling decreases, indicating a sharper thermal gradient in tissue for these exposures.

In vivo AMF safety studies were conducted to evaluate the extent of surrounding soft tissue damage as a function of AMF exposure parameters, and to demonstrate the ability to automatically shut off treatment using the acoustic emission detection method developed in this study. In mice, the boiling acoustic emissions were stronger than those observed for the tissue-mimicking gel, leading to a higher sensitivity of the system. This is probably due to the higher water content around the ball in the muscle. Fig 8A–8D show hematoxylin and eosin (H&E)-stained sections of the muscle at the location of the implanted ball (represented by a cavity in the images). An inflammatory response from the surgical procedure in control animals was observed on the implant-tissue boundary at 7 days (Fig 8A), but had largely disappeared after 14 days (see S1 Fig). Fig 8B–8D show tissue damage of the surround thigh muscle in mice exposed to AMF with three different powers (190, 800, 4300 W), respectively. It is evident that increasing the AMF power (which reduced the time required to reach boiling) resulted in a reduced radius of localized thermal damage around the cavity, indicated by the dashed line in the images. The measured radii of the thermal damage were 3.05 ± 0.33 mm for the 190 W exposures, 1.33 ± 0.30 mm for the 800 W exposures, and 0.60 ± 0.21 mm for the 4300 W exposures. Within the region of thermal damage, connective tissue exhibited a homogeneous appearance, and vascular channels were distended with coagulated blood contents. Additionally, the muscle fibers were fragmented and contained scattered contraction bands, and the endomysium was expanded, leading to separation of muscle fibers.

The in vivo experiments showed that thermal damage around the metal ball was dependent on power/heating duration, with the radius decreasing for higher powers/shorter times. We chose to heat the ball to the point of boiling to achieve a consistent surface temperature in each group. The thermal dose (CEM43) on the ball surface were 6.0×10^19^ (200W, 182.5s), 4.2×10^17^ (800W, 6.4s), and 1.1×10^10^ (4300W, 1s) based on the temperature curves obtained in Fig 3. For comparison, we also conducted Finite Element numerical simulations by using COMSOL software analysis with the same conditions as the experiment (surface boiling achieved for different heating times), and characterized the radial distance of the 240 CEM43, 80 CEM43, and 16 CEM43 contours (Fig 8E and 8F). CEM43 is a convenient metric that factors temperature and time and allows comparison of different thermal exposures [18]. 240 and 80 CEM43 represent upper and lower thresholds for irreversible thermal damage in muscle [19,20]. 16 CEM43 indicates a threshold thermal dose for initial bone damage. The tissue damage is relatively reversible between 80 and 240 CEM43 and becomes irreversible once beyond 240 CEM43. Fig 8F shows the radial distance from the surface of the ball to the 80 and 240 CEM43 boundary in muscle as a function of the time required to reach boiling on the surface of the ball. The time to reach boiling ranged from approximately 1 to 300 seconds. As shown, there is a rapid reduction in the radius of thermal damage as the time to boiling decreases. For exposures lasting on the order of a second, the soft tissue damage is less than 1 mm from the surface. Furthermore, the distance between the 240 and 80 CEM43 contours is also reduced, indicating a sharper thermal gradient in tissue at short exposure times. This points to the benefits of employing high-power AMF exposures to achieve heating the metal surface as quickly as possible in order to preserve the maximum amount of adjacent soft tissue. These trends, and the distance to the 240 CEM43 contour reasonably approximate the distances observed in the mouse study, factoring in the variability in those tissue sections due to distortion and shrinkage during fixation.

**Fig 9:**
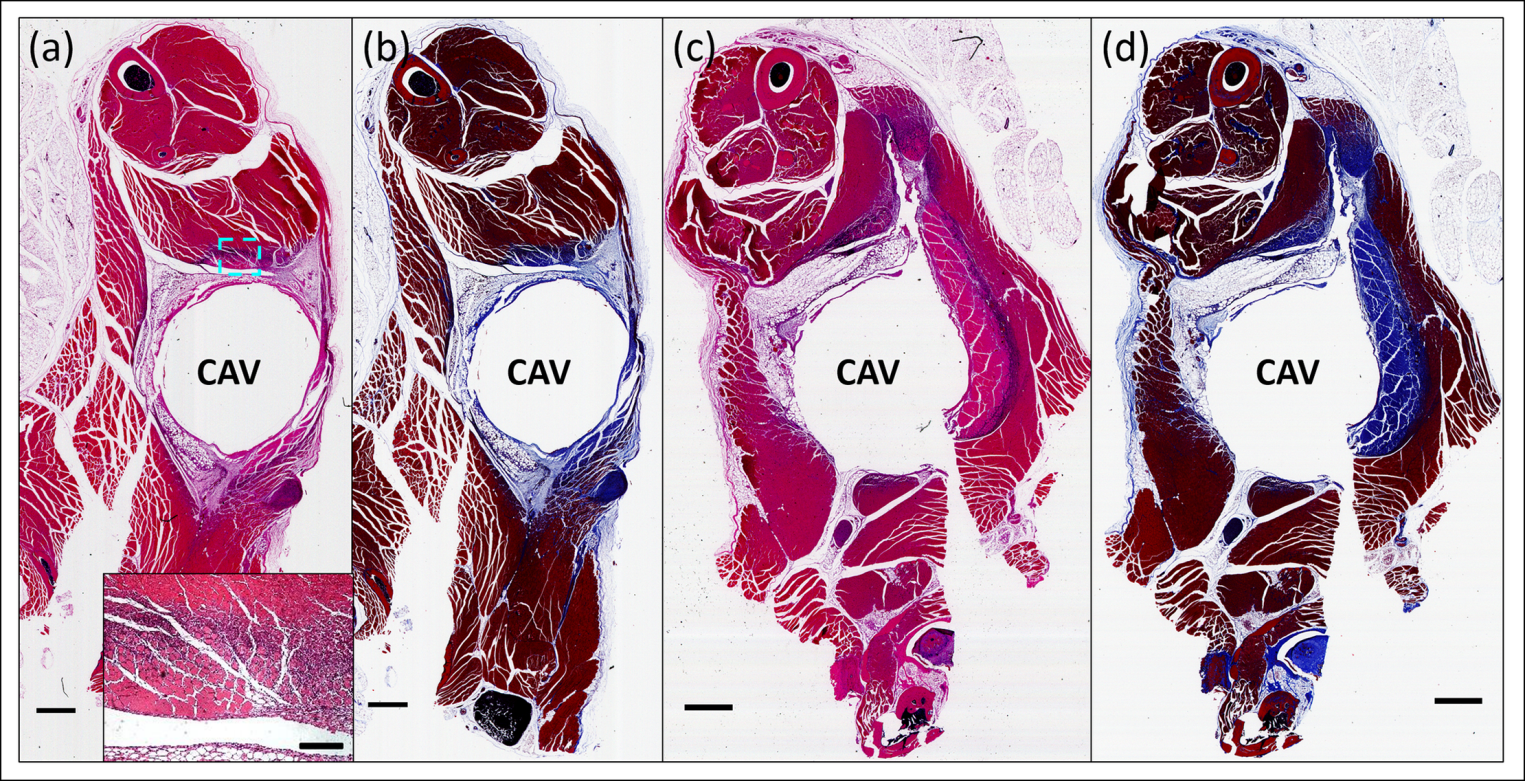
Comparison between H&E- and Masson’s trichrome-stained sections showing tissue thermal damage. A mouse 7 days after an 800 W AMF exposure lasting 4 seconds exhibits a circumferential pattern of damage in both (a) H&E staining and (b) Masson’s trichrome staining. In H&E-stained section (a), robust regenerative activity is seen at the edge of the thermal damage boundary (purple area). To representatively show the details, a high-magnification image of the area indicated by a blue-dashed box in was obtained and showed as the inset with the scale bar of 200 μm. In Masson’s trichrome-stained section (b), the blue area indicates necrotic muscle fibers, matching that seen in H&E-stained sections. (c,d) A mouse 7 days after a 4300 W AMF exposure shows similar findings. In all cases, the transition from damaged to normal muscle is abrupt and is best visualized on Masson’s trichrome stain. Scale bars represent 1 mm.

Fig 9 shows both H&E- and Masson’s trichrome-stained sections of the implant area in mouse thigh tissue seven days after AMF exposures of varying power (800 W, Fig 9A and 9B; 4300 W, Fig 9C and 9D). At each AMF power, H&E-stained sections (Fig 9A and 9C) and Masson’s trichrome stained-sections (Fig 9B and 9D) reveal almost identical zones of thermal damage in the surrounding muscle, although the damaged regions are more evident on Masson’s trichrome sections. Seven days after the AMF exposure, myocytes within the damaged region are necrotic, and a rim of regenerating myofibers surrounds the well-defined area of thermal damage. Fibroblastic proliferation and macrophage infiltration of blood vessels are apparent within and surrounding the damaged area, indicating an ongoing repair response (see the inset of Fig 9A). Trichrome staining confirmed the findings in H&E staining, with more obvious boundaries between necrotic and viable muscle.

### 2.8 A human-scale AMF system

**Fig 10:**
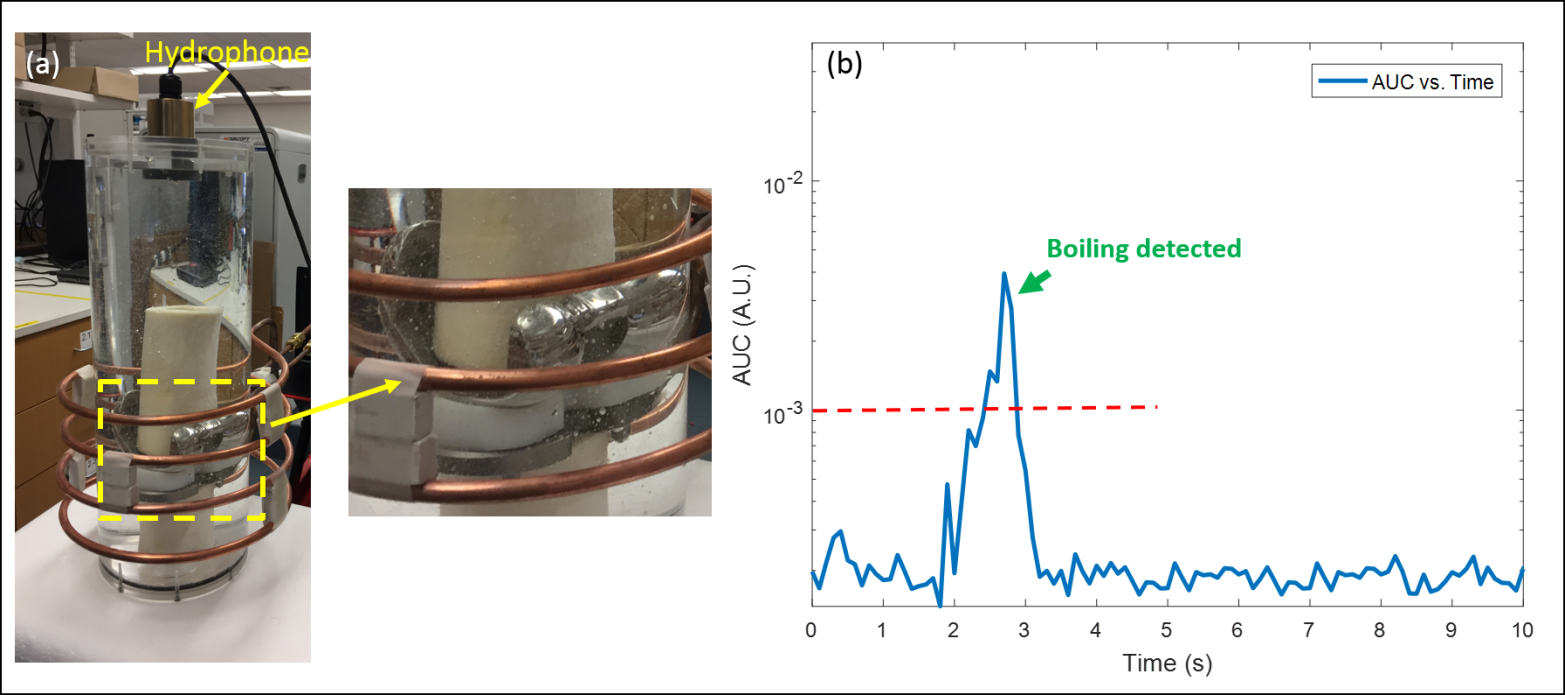
Boiling detection of a human-sized knee phantom. (a) A human-scale AMF system set up, including a human-sized knee implant, a customized coil which fits the knee size, and a hydrophone on top. (b) Sound signal during AMF-induction heating. The red dashed line indicates boiling signal detection threshold (0.001). The figure demonstrates that efficient heating on the human knee implant was achieved with the customized AMF system, and acoustic emissions related to boiling were successfully detected. Power: 4300 W.

A scaled-up AMF system was developed for delivering exposures to a human knee implant. As shown in Fig 10A, a prosthetic knee was placed in a cylinder containing a tissue-equivalent phantom [21] attached with a hydrophone on top. The measured acoustic emissions are shown in Fig 10B. The red dashed line indicates the boiling AUC threshold. Herein, the threshold was modified to 0.001 due to the different background noise level (since this is a different system). The time for the knee required to reach boiling was 2.5s with a 4300 W AMF exposure.

## 3. Discussion

We recently proposed a non-invasive approach for biofilm eradication on metal implants by employing high-frequency alternating magnetic fields [14]. High-frequency AMF induces rapid heating on the outer metal surface of an implant which can be bactericidal to biofilm. A legitimate concern with respect to the safety with this method of treatment is potential thermal damage to surrounding tissues. A remote temperature monitoring method is essential and of high significance to this new technology to mitigate this potential risk. In this study, we explored the potential utility of acoustic emissions associated with boiling at the interface between metal implants and the surrounding environment as a wireless indicator of that some points of the implant surface have been heated to approximate 100°C. Experiments conducted with implanted fiber-optic sensors verified that this acoustic signal was detectable with a remote hydrophone when the metal surface reached boiling temperature. This relationship was consistent and was not affected by the power of AMF exposures, surrounding media, location within the AMF transmitter, or the geometry of the implant. The main limitation observed was when the implant was embedded in oil (a mimic of fatty tissue), where the acoustic emissions were not detected at 100°C, due to the higher boiling temperature of oil.

Boiling signatures were reliably identified within the frequency band between 400 and 1000 Hz in both tissue-mimicking phantom and in vivo studies, and an area under the curve (AUC) was calculated across this band to represent the intensity of acoustic emissions. The AUC ratio between the boiling and background signal within this frequency band was greater than 10. Based on the large SNR, a boiling control algorithm was developed that was used to shut off AMF power when the AUC was beyond a certain threshold, e.g. 10 times of background level. This algorithm was very robust due to the unique boiling signatures and large SNR for all the experiments conducted in this study. However, the selection of an integration of signal across a frequency band is a relatively simple approach, and more sophisticated detection algorithms might be better able to separate environmental noise from boiling. Also, the boiling signatures may have some relationship with the surrounding media. Although the characteristic frequency band between 400–1000 Hz worked well for the gellan-based tissue-mimicking phantom and the thigh muscle of mice, 400–3000 Hz worked best for a Carpobol hydrogel, and 400–1500 Hz for an ASTM phantom. Therefore, future work to refine the acoustic control algorithm should result in even greater robustness and reliability. Note that, the choice of frequency bands discussed here corresponds to the particular hydrophone used in this current study. The response frequency band of this hydrophone may not be best for the boiling detection. It is worth exploring boiling detection using hydrophones with different response frequency bands and also investigate the appropriate characteristic band for each hydrophone in the future.

The time required to reach boiling was strongly dependent on the AMF power, position inside the coil, and the geometry of the metal implants. It will also have a strong dependence on the composition for implants of the same geometry. This variability in heating efficiency due to multiple factors further emphasizes the importance of having a remote signal that indicates a known thermal endpoint has been reached. Applying pre-calculated or fixed power & duration AMF exposures is unlikely to produce a repeatable thermal effect on different metal implants, or even the same metal implant if positioning inside the coil varies slightly. However, by using the remote acoustic detection, there would be confidence that a defined surface temperature was not exceeded on the implant irrespective of these different factors. In this study, the metal used was 316 stainless steel, which only represents a subset of the metals used in medical implants. However, this acoustic sensing technique can also be applied on implants with other materials such as titanium alloys or CoCr alloys, although the heating may be either faster or slower compared with stainless steel based on their electrical conductivity/resistivity and response to magnetic induction. For example, Titanium has an electrical resistivity of 0.0000554 Ω cm while 316 stainless steel has a slightly larger electrical resistivity of 0.0000740 Ω cm, so Titanium will have a slightly stronger induction heating effect. Nonetheless, the safety can be preserved for metal implants with all these different materials as the system will shut off the power as long as it detects the boiling regardless of the heating speed.

Both in vitro and in vivo studies demonstrated that with more power deposited on the metal implant, less time was required for the metal implant to reach boiling temperature. At 4300 W, approximately 1 second was required to heat the metal ball bearing surface to 100°C. The ability to achieve high surface temperatures very rapidly could result in a safer treatment by reducing the amount of time for heat to conduct into surrounding tissues. Further, since only the outer surface of the metal implant is heated due to the skin effect, the inner metal mass of the implant can serve as a heat sink. These effects should lead to a lower thermal dose and hence reduced tissue damage at higher power, which was observed in the in vivo experiments described in this study. Acoustic emissions were reliably detected in every mouse that received an AMF exposure except one, and upon sacrifice it appeared to be located in a fat pad. Both H&E-stained and Masson’s trichrome-stained sections revealed that the tissue thermal damage adjacent to the heated metal is very localized and can be confined to less than a millimeter around the implant.

Finally, this method of remotely detecting boiling was successfully scaled up to a human prosthetic knee implant. Using a human-scale system, acoustic emissions indicating boiling were measured within 2.5 s for a 4300 W AMF exposure. This ability to measure surface boiling across a wide range of sizes of implants confirms the robustness and applicability of this measurement technique. Another important benefit of this acoustic sensing technique is the ability of real-time monitoring. Since sound travels at 1500 m/s in soft tissue, boiling can be detected within microseconds, and AMF power can be shut off to avoid heating beyond this level. However, as we have mentioned in our previous report (14), the heating of the complex 3D objects is not uniform which will lead to that some areas were underheated while some points of the surface have been heated to boiling temperature. More studies are currently undergoing to improve that.

One potential limitation for this acoustic technique is the use of a hydrophone, which requires some fluid coupling between the implant and the receiver, however, clinical devices may have fluid circulating around the targeted limb to protect skin and surrounding tissue from thermal damage. Also, the importance of positioning the hydrophone was not investigated in detail in this study, and should be further evaluated. Finally, EMF interactions between the hydrophone and AMF system are an important consideration, and appropriate shielding is required to avoid cross-interference. Another limitation is this acoustic sensing system can only indicate the boiling temperature of the implant surface (approximate 100°C). Other temperatures either below or above the boiling temperature cannot be controlled with this technique, e.g., 60 or 80°C, even though they may be more efficient and safer for the AMF therapy. In addition, in this study it is not clear that whether the surrounding tissues will be altered such that boiling can no longer occur after some exposures. Lastly, this study only tested the feasibility of the acoustic detection method for metal implants adjacent to soft tissues. Whether this method would work on implant surfaces embedded in bone (i.e., intra-medullary stems or rods) was not evaluated. However, we predict that there would be enough tissue water to produce a boiling signal, but the acoustic propagation/attenuation of this signal through bone is unknown. Although promising, further studies are required to determine whether acoustic emissions during the AMF treatment can serve as a remote and robust tool for the safety control.

## 4. Conclusion

In this study, we have demonstrated the successful and consistent detection of acoustic emissions associated with boiling at the metal implant/tissue interface during AMF exposures. We believe this acoustic measurement can be used as a robust and wireless safety mechanism in AMF biofilm treatment. A proof-of-concept AMF system for in vitro and in vivo studies has been developed. Boiling signals were reliably identified in both phantom and mouse studies, and also in a human-scale AMF system heating a human knee implant. Short duration, high-power AMF exposures resulted in the least amount of thermal damage to surrounding tissues, and may be a safe way to deliver this treatment.

## 5. Methods

### 5.1 AMF system setup

A custom-built solenoid was used to expose metal objects implanted in a phantom or mouse to an alternating magnetic field. A four-turn solenoid coil (5 cm diameter, 3.5 cm length, 0.9 cm pitch, 0.85 μH inductance) was constructed from copper tubing (OD: 3/16”, ID: 1/8”, P/N 8950K522, McMaster-Carr, Douglasville, GA, USA). The solenoid was connected to a water cooling system (Accel 500 LC, Thermo Scientific, Waltham, MA, USA) to maintain the coil temperature at 15°C. A 520 kHz driving signal produced by a function generator (33250A, Agilent Technologies, Santa Clara, CA, USA) was amplified through an RF amplifier (1140LA, Electronics & Innovation, Rochester, NY, USA), and transmitted through an LC-matching and resonant tank circuit which included the working coil. The system was capable of delivering up to 800 W to the coil. For the acoustic detection sub-system, a submersible hydrophone (SQ26-10, Cetacean Research Technology, Seattle, WA, USA) was utilized to collect the sound signals emitted by the metal implant. The sample tube was connected to a cylindrical tube filled with water for efficient sound propagation between the implant and the hydrophone. For the in vivo studies, the mice with ball implants was placed in the sample tube coupled with warm water to maintain the body temperature. The acoustic signals were digitized with a data acquisition card (DAQ, sampling rate: 48000 Hz, sampling time: 92 ms, repetition frequency: 10 Hz, M-Audio B9597911, Cetacean Research Technology, Seattle, WA, USA). The function generator and data acquisition were controlled by a custom-written LabVIEW (LabVIEW 2015, National Instruments, Austin, TX, USA) program on a personal computer (PC). This system was used for all the experiments in this study except those with a human knee implant.

### 5.2 Tissue-mimicking phantom

Gellan gum-based tissue-mimicking phantoms were fabricated according to a previously described protocol [22]. Herein, the procedure was modified and briefly described. First, 12.5 ml propylene glycol (PPG, Froggys Fog, Columbia, TN, USA) and 63.5 ml degassed water were mixed in a beaker. Then the mixture was heated up by placing the beaker on a hotplate stirrer (H4000-HS, Benchmark Scientific Inc., Edison, NJ, USA) with stirring. 1 g gellan-gum powder (Gelrite, CP Kelco, Atlanta, GA, USA) was added slowly into the mixture while stirring, to avoid clumping. 0.25 g sodium propionate (P1880-100G, Sigma-Aldrich, St. Louis, MO, USA) was introduced into the mixture to prevent bacterial growth, following by 24 ml 0.9% NaCl solution to adjust the electrical conductivity to that of muscle. Once the solution was 85°C and the solution was transparent, it was cooled to approximately 60°C, and poured into a 50 ml centrifuge tube. The tube contained a 4.8 mm 316 stainless steel ball (P/N 96415K73, McMaster-Carr, Douglasville, GA, USA) with a fiber-optic temperature sensor (PRB-G40-2M-STM-MRI, Osensa Innovations, Burnaby, BC, Canada) epoxied onto its center with a thermal compound (ASTA, Arctic Silver Inc, Visalia, CA, USA). Two hours later, the tissue-mimicking phantom with the embedded ball bearing was solidified and ready for use.

### 5.3 Acoustic and temperature detection in tissue-mimicking phantoms

During AMF exposures, the acoustic signals produced from the heated metal implant were monitored with the hydrophone and the temperature of the ball was recorded with the fiber-optic temperature sensor. The fiber-optic sensor was connected to a temperature transmitter (FTX-300-LUX+, Osensa Innovations, Burnaby, BC, Canada) capable of recording the temperature at a rate of 10 Hz. AMF exposures at 200, 250, 300, 400, 800, and 4300 W were performed with the tissue-mimicking phantom. For the 4300 W experiments, a commercial AMF system (EASYHEAT LI 8310, Ambrell Corporation, Scottsville, NY, USA) was used since the maximum power of the custom-built system was 800 W. The coil used with the commercial system was similar in dimensions to the one connected to the custom-built AMF system, but the frequency of transmitted signal was slightly lower (173 kHz) due to limitations of the commercial system.

### 5.4 Boiling control threshold selection

Fast Fourier transform (FFT) was performed on the detected acoustic signals. Based on the measured frequency spectrum, a specific characteristic band was selected between 400–1000 Hz, and the area under the curve (AUC) within this band was calculated. The AUC was measured as a function of time during AMF exposures to represent the strength of the emitted sound signal. A threshold indicative of surface boiling at the implant was selected to be 10 times the background signal. The AMF system was shut off whenever the detected AUC was greater than this threshold. In this study the AUC threshold was selected as 0.003 for the phantom and mouse studies, and 0.001 for the human-scale AMF system.

### 5.5 Acoustic and temperature detection in different media

In these experiments, the influence of the medium surrounding a metal implant on the acoustic emissions was investigated. Three different media were investigated, including the tissue-mimicking phantom described above, a hydrogel with adjustable viscosity, and oil. In all cases, the embedded implant was the metal ball with the attached fiber-optic sensor as previously described. In the oil study, 40 ml motor oil (0W-30, Mobil, Dallas, TX, USA) was added to the 50 ml centrifuge tube. In the hydrogel study, 40 ml of hydrogel was added to the tube. Briefly, the hydrogel preparation involved adding 1% (w/v) of acrylate crosspolymer powder (Carbopol Ultrez 21 Polymer, Lubrizol, Cleveland, OH, USA) to degassed, deionized water at room temperature. After proper mixing, the solution was poured into a 50ml conical tube. The viscosity of the gel could be adjusted by changing the pH value. Different amount of 18% NaOH solution was added into the conical tube to form phantoms with various viscosities. The power applied for all these experiments was 800 W.

### 5.6 Influence of position on time to boiling

For these experiments the stainless steel ball was inserted in the centrifuge tube filled with 40 ml of hydrogel with a viscosity similar to water. 800 W AMF exposures at 520 kHz were performed with the ball at different axial and lateral positions within the coil, and the time to boiling was recorded at each position. In the axial direction, the ball was moved to locations 5, 10, 15, and 20 mm away from the center. In the lateral direction, the ball was moved to locations 6 and 12 mm away from the center.

### 5.7 Boiling detection for implants with different geometries

The influence of implant geometry on the acoustic emissions was evaluated using stainless steel balls, washers, and rings. The mass of the metal ball, ring and washer is 0.5, 2.1, and 1.9 g, respectively. The ring has the outer diameter of 0.75 inches with a wall thickness of 0.035 inches and the height was 0.2 inches. The inner and outer diameters of the washer were 0.174 and 0.750 inches, respectively. The thickness of the washer may vary from 0.033 to 0.047 inches. The washer and the ring were placed inside the coil in perpendicular to the magnetic field. All of the objects were placed in a 50 ml centrifuge tube and surrounded by either low viscosity hydrogel or water. The powers used for these experiments were 800 W for the ball and 650 W for the washer and ring. The AUC as a function of time was recorded for each implant.

### 5.8 Boiling detection of a human knee implant

The feasibility of detecting acoustic emissions due to surface boiling in a human knee implant was evaluated using a prosthetic knee embedded in a tissue-mimicking phantom, and a commercial AMF generator. The human prosthetic knee has the dimension of 8×6×11 cm in terms of its bounding box. The mass of the femur and the tibia components is 300 g and 150 g respectively. A four-turn solenoid coil with a parallel structure (e.g., two turns were connected with another two in parallel to achieve an inductance of 0.7 μH) was constructed from copper tubing. The parallel design was implemented to maintain an inductance within the range of the generator matching electronics. The diameter of this coil was 16.5 cm and the length was 9.5 cm. A commercial induction heating system capable of delivering up to 10 kW was used as the power supply (EASYHEAT LI 8310, Ambrell Corporation, Scottsville, NY, USA). As shown in Fig 10, the hydrophone was placed above the knee phantom, coupled with a fluid path. The boiling threshold in this setup was selected at 0.001.

### 5.9 In vivo acoustic detection and safety evaluation

A preclinical study was performed to evaluate the feasibility of detecting acoustic emissions associated with implant-tissue interface boiling, and the tissue damage produced adjacent to implants exposed to AMF. These experiments were approved by the Institutional Animal Care and Use Committee (IACUC) at UT Southwestern Medical Center (approval number: 2016–101572), and conformed to the National Institutes of Health’s PHS Policy on the Humane Care and Use of Laboratory Animals. All researchers involved in the animal study received animal care and handling trainings from the Animal Resource Center at UT Southwestern Medical Center.

Female Swiss Webster mice (30–40 g, mean = 32.5 g, n = 54) were anesthetized using an IP injection of ketamine (80 mg/kg) and xylazine (5 mg/kg), and a subcutaneous injection of Buprenorphine (slow release) (0.6 mg/kg) was administered for pain control and to minimize distress due to pain. The fur on the thigh was shaved and depilated, and the exposed skin was sterilized with providone iodine and alcohol. An incision was made on the thigh to create a pocket in muscle deep enough to insert a 4.8 mm diameter stainless steel ball. The incision was closed with coated VICRYL 5–0 absorbable suture (J391, Ethicon, Somerville, NJ, USA), and animals were recovered for 7 days. Animals were then anesthetized with isoflurane (1–3%, mixed with oxygen), secured to a platform, and inserted into a solenoid coil such that their thigh with the implanted ball was approximately centered along the length of the custom-built solenoid coil. An AMF exposure was delivered until boiling was detected acoustically, indicating the same final surface temperature on the ball in all animals. One group of animals (n = 5) received AMF exposures with a low power (190–260 W) and long duration (220–300 s). Another group (n = 14) received medium powers (800-900W) which achieved boiling in a much shorter time (4–12 s). Some of the animals receiving the 800 W AMF exposures (n = 6) were survived for an additional 7 days to observe the delayed tissue effects in the muscle. The third group (n = 10) received high power AMF exposures (4300 W) with an even shorter time to boiling (1–2 s). Half of the animals in this group were survived for an additional 7 days to observe the delayed muscle tissue effects. Control groups of animals receiving sham AMF treatments were sacrificed 48 hours (n = 2), 7 days (n = 2) and 14 days (n = 2) after implantation to match the exposed animals in terms of handling and insertion in the coil. The remaining animals (n = 16) were used to develop the surgical technique, calibrate powers, or refine the histological processing method so that consistent outcomes can be achieved. In this group, animals were euthanatized immediately after the experiment by administering 240 mg/kg Sodium Pentobarbital through an IP injection. Some of the animals (n = 3) died during surgical implantation due to anesthetic complications. Survival animals were returned to ventilated, non-conventional housing in the animal facility. They were provided with clean bedding, food, water, and enrichment (red huts and nestlets) and were monitored daily by laboratory personnel for 72 hours post operatively for any post-operative complications (wound dehiscence, infection, refusal to utilize the surgical limb) as well as failure to thrive (i.e. lethargy, hunched posture, anorexia, rough coat). They were also observed daily for the duration of their life by the UTSW Animal Resource Center staff for any of the aforementioned signs. If any of these signs were observed, or if the animal reached the endpoint of the experiment (7 days for acute studies or 14 days for delayed tissue effects assessment), a lethal dose of Sodium Pentobarbital (240 mg/kg) was administered IP to achieve humane euthanasia immediately.

Upon sacrifice, the leg with the implant was harvested and fixed in formalin for 5–7 days. The tissue was then transferred to a decalcifying solution (Cal-Rite, 5501, Thermo Fisher Scientific, Waltham, MA, USA) for 14 days to decalcify the tibia and femur. Once decalcified, the ball was carefully removed from the leg (leaving behind a cavity), and the limb was processed for paraffin histology. H&E- and Masson’s trichrome- stained tissue sections were obtained through the implant cavity to evaluate the radial extent of thermal damage around the AMF effecting ball. The histology was reviewed by two pathologists, blinded to the exposure conditions.

### 5.10 Microscopy

Low-magnification scans of all histologic preparations were obtained using a PrimeHisto XE histology slide scanner and Histoview 1.00 acquisition software (PacificImage Electronics, Torrence, CA). High-magnification photomicrography of thermal damage pathology was carried out on a Leica DM2000 photomicroscope equipped with bright-field illumination and an Optronics Microfire digital CCD color camera. High magnification images were captured using PictureFrame 3.0 acquisition software (Optronics,Inc. Goleta, CA, USA) and processed with Adobe Photoshop.

### 5.11 Finite Element Analysis (FEA) simulations

Finite-element bioheat transfer simulations were performed in COMSOL (Comsol v5.2, Comsol multiphysics, Stockholm, Sweden) to investigate the influence of different AMF exposure conditions on the amount of heating and thermal damage in surrounding soft tissue. The simulation model matched the in vivo mouse studies presented in this study comprising a 4.8 mm stainless steel ball implanted in mouse thigh muscle, and exposed to AMF.

#### Model geometry and materials

A simplified 2D axisymmetric model of a 4.8 mm diameter metal ball inserted in a cylindrical muscle volume and surrounded by water was generated. The ball was assigned material properties of 316 stainless steel, while the water and muscle domains were assigned material properties from the built in COMSOL database and the ITIS tissue library, respectively. The material properties were summarized in S1 Table. The water had an initial temperature of 35°C and the tissue 37°C based on the measured values in the in vivo experiments.

#### Physics modeling

The tissue was modeled using the Biological Tissue module, which includes the heat transfer attributable to perfusion, the values for which were taken from the ITIS database [23]. The ball and water were modeled using the standard heat transfer module, with a boundary heat source on the surface of the ball used in place of direct AMF heating (which was parametrically adjusted to simulate different times to boiling). To accurately represent the acoustic detection implemented in the experiments, the boundary heat source was linked to a probe such that when the maximum surface temperature of the ball bearing reached 100° C the heat source was turned off, and the ensuing cooldown was simulated for a total simulation duration of 20 minutes. Thermal tissue damage was modeled using the cumulative equivalent minutes at 43°C units (CEM43) based on the model proposed by Sapareto and Dewey [18]:

$$CEM43=\int_{0}^{t}r^{43-T},r=\left\{ \begin{array}{c} 0.25,T<43\\ 0.5,\;T\geq 43 \end{array}\right.$$

Once the thermal dose distribution in tissue was determined (after the 20 minutes of heating and cooling) the 240, 80, and 16 CEM43 surfaces were identified and the distance from the surface of the ball to these locations measured.

#### Meshing and solving

The model was meshed using the automated “extremely fine” mesh in COMSOL, with a minimum of 60 elements on the surface of the bearing. The applied power to the surface of the bearing was stepped from 17 kW/m^2^ (total power = 1.4 watts) to 200 kW/m^2^ (total power = 14.2 watts), which corresponded to times to achieve boiling of roughly 280 seconds and 1 second respectively. Solving took approximately 10 minutes on a Windows 7 PC with a 6 core Intel i7 processor and 128 GB of RAM.

## Supporting information

S1 Fig H&E-stained sections through the thighs of mice after 14 days of the surgical procedure (Scale bar = 1 mm). (TIF)

S1 Table Material properties used in finite element simulations. (PDF)
