# Supplementary figures and images for: Remote acoustic sensing as a safety mechanism during exposure of metal implants to alternating magnetic fields
Source: PLoS One. 2018 May 10;13(5):e0197380. doi: 10.1371/journal.pone.0197380 (PMC5944992; doi:10.1371/journal.pone.0197380)

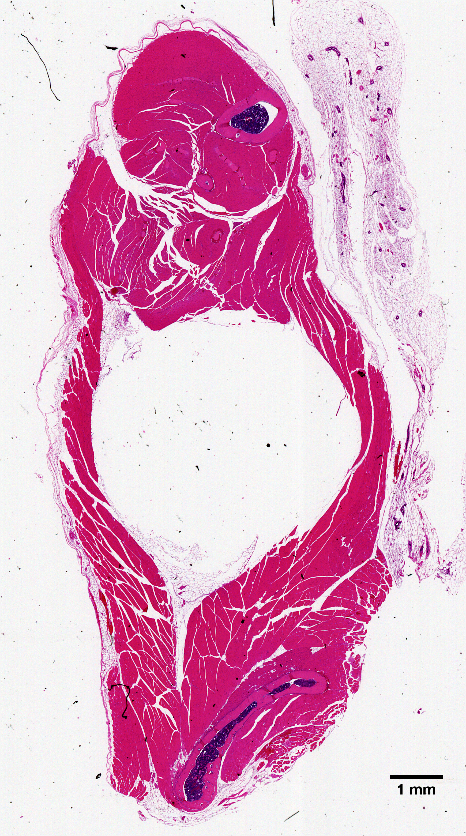

Supplement: S1 Fig — (TIF) [file pone.0197380.s001.tif]
