# Supplementary material for: Remote acoustic sensing as a safety mechanism during exposure of metal implants to alternating magnetic fields
Source: PLoS One. 2018 May 10;13(5):e0197380. doi: 10.1371/journal.pone.0197380 (PMC5944992; doi:10.1371/journal.pone.0197380)
Supplement: S1 Table — (PDF) [file pone.0197380.s002.pdf]

S1 Table. Material properties used in finite element simulations

	Muscle	316 stainless steel
**Heat Capacity**	3421[J/(kg*K)]	0.5[J/(g*degC)]
**Density**	1090[kg/m^3]	8[g/cm^3]
**Thermal Conductivity**	0.49[W/(m*K)]	16.3[W/(m*K)]
**Electrical Conductivity**	0.599[S/m]	1/0.000074[ohm*cm]
**Perfusion**	37[ml/(kg*min)]	
